# Development of an eco-friendly geopolymer mortar using slag and fly ash with high bentonite content for thermal and environmental applications

**DOI:** 10.1038/s41598-024-76780-5

**Published:** 2024-11-05

**Authors:** Aya allah M. Ebrahim, Doaa A. Ahmed, Reham Abu-Elwafa

**Affiliations:** 1 Chemistry Department, Faculty of Women for Arts, Science and Education, Ain Shams University, Cairo11757, Cairo, 11757 Egypt; 2 Housing and Building National Research Center, 87 El Buhouth St, Giza, 1770 Cairo Egypt

**Keywords:** Ternary geopolymer mortar, Slag, Fly ash, Bentonite, Adsorption, Heat resistance, Environmental sciences, Chemistry, Materials science

## Abstract

The construction industry is exploring the use of low-cost waste materials to create eco-friendly geopolymer mortar binders. Our study aims to develop various environmentally friendly geopolymer mortar mixes for thermal and adsorption applications using natural materials like bentonite and industrial by-products such as ground-granulated blast furnace slag and fly ash. Ternary geopolymer mortar pastes are prepared using equimolar amounts of slag (GBFS) and fly ash (FA), with 6%, 8%, 10%, and 12% weight of bentonite (BC) from the total geopolymer weight to study the bentonite replacement effect. The prepared mortar are tested for their physico-chemical, mechanical, adsorption, and thermal stability properties (300 °C to 900 °C). The adsorption behavior of eco-friendly geopolymer mortar mixes against crystal violet dye in aqueous solutions is also identified. The study found that adding 6% bentonite to the slag/fly ash-based geopolymer mortar mix yielded the highest mechanical characteristics. Moreover, all the ternary geopolymer mortar mixes exhibited excellent thermal stability up to 900 °C. In adsorption study, the results indicated that the mortar mixes had excellent capacities and adhered well to the Freundlich isotherm model, suggesting potential applications in treating wastewater. Using bentonite in slag/fly ash geopolymer mortar offers a sustainable, cost-effective, and heat-resistant alternative to traditional cement binders.

## Introduction

With the expansion of the population and the need for infrastructure, there is an annual rise in demand for the manufacture of OPC. Natural limestone supplies are running out during OPC production, and there is also a significant embodied energy consumption. The emission is a significant environmental concern as well as a significant amount of CO_2_ which is regarded as one of the most important causes of global warming. It is thought that the production of cement releases roughly 6% of the gases that contribute to global warming^1,2^. The use of waste products from industries as an OPC substitute was deemed to be useful for the production of mortars and concrete to alleviate such issues^3,4^. A crucial aspect of sustainable development is the recycling of waste into usable building materials. In this sense, geopolymer was crucial as an unusual binding agent for the creation of concretes^5,6^. These concrete made from waste resources exhibit enhanced mechanical performance and durability qualities^7^. Geopolymer is accomplished by polymerizing alumina and silica in a substance that is a rich source of aluminosilicates activating within an alkali activator solution^8^. The majority of aluminosilicate materials are mined from the earth or created by industry as wastes, such as calcined kaolin or metakaolin (MK), palm oil fly ash (POFA), fly ash (FA), silica fume (SF), rice husk ash (RHA), bentonite (BC) and granulated blast furnace slag (GBFS)^9,10^. The amount and type of alkaline solution, the molarity of the alkaline solution, the fineness and shape of the binder materials used in the manufacturing of geopolymer mortar/concrete, as well as other aspects, affect the strength and durability of geopolymer mortar/concrete^11,12^. Furthermore, it has been found that early strength development is poor when employing a single binder; however, geopolymer mortar’s efficiency is increased when two or more binding components are combined. The rate of hydration is accelerated by adding ternary additional cementitious material to the geopolymer, which further enhances compactness and reduces weight loss while improving strength and durability attributes^13,14^. Moreover, multi-material binders are recognized to possess characteristics that make them particularly well-suited for enhancing the early and normal strengths of mortars and concrete, as well as other associated qualities^15^. Nevertheless, single-material geopolymer binders made from clay, slags, or ash show issues such as surface cracks and inadequate early strength ^16^.

The abundant availability of raw clay and the active components produced by the decomposition of clay minerals due to thermal treatment, either naturally or artificially, have made the use of nano-clay or calcined clay in concrete an effective replacement for supplementary cementitious materials^17^. Bentonite clay (BC), which is overabundant in parts of Asia and Africa, is one of the interesting and prospective alternatives^18^. BC is a smectite-derived clay mineral with a high montmorillonite concentration. BC has a huge surface area and a significant concentration of calcium and sodium ions. In the presence of water, bentonite can bind the sand grains in concrete to form a plastic paste, creating a workable combination^19^. By combining BC with FA, the concrete’s compressive strength has risen by 10% while also exhibiting improved thermal properties^20^. It was observed that^21^ replacing 15% of the BC by mass of OPC can increase compressive and tensile strength while reducing chlorine migration into natural aggregate concrete (NAC) and recycled concrete aggregate (RCA). Moreover, in comparison to ordinary concrete, the use of BC (up to a 20% substitution of OPC) in conjunction with metakaolin or FA has produced good sulfate resistance, compressive strength, and microstructural characteristics^22^.

Granulated blast furnace slag (GBFS), is very appropriate as a geopolymer binder due to its amorphous form and CaO, SiO_2_, and Al_2_O_3_ enrichment and also exhibits pozzolanic properties^23^. FA use in geopolymer concrete provides several advantages, including increased workability, decreased dryness shrinkage, improved resistance to chemical assault, decreased corrosion of steel reinforcement, and decreased hydration heat^24^. The mechanical and microstructural properties of GBFS containing FA geopolymer were improved and this is due to the overproduction of calcium derived from GBFS^25^. The mechanical strength of FA combined GBFS was studied^26^, which was shown to withstand a compression strength of 50 MPa at the age of 28 days.

Dyes and other organic pollutants in industrial effluents are one of the primary causes of contaminated water sources. To reduce dye pollution, a viable remediation strategy must consider the features of dyes, including their identification, quantification, and classification. For instance, cationic dyes like crystal violet (CV) can cause negative health effects in humans. Furthermore, the presence of aromatic rings in their structure makes these organic pollutants potentially carcinogenic^27^. Water pollution rehabilitation is a field that has seen the development and application of numerous treatment methods, including chemical, biological, and physical ones^28^. The adsorption process is a simple, cost-effective, and efficient method of purifying water that can be used with a wide range of adsorbents and doesn’t produce hazardous sludge or additional wastes^29^.

Although there is no existing literature on preparing geopolymer mortar using a three-component mix of slag, fly ash, and high bentonite content, this research aims to develop a new geopolymer mortar binder with cost-effective materials. It addresses environmental concerns while enhancing performance and minimizing costs. Our goal is to create eco-friendly geopolymer mortar mixtures by incorporating natural components like BC and industrial waste such as GGBFS and FA. This study aims to improve the strength, expansion, and thermal stability of the ternary geopolymer paste by incorporating sand as a fine aggregate. We prepared geopolymer mortar pastes with different amounts of BC (6%, 8%, 10%, and 12% by weight) using fly ash, slag, sodium silicate, and sodium hydroxide in a 2.5:1 ratio as alkali activators. The hydration properties of these environmentally friendly ternary geopolymer mortar mixes were examined after water-curing for periods ranging from 3 to 28 days at room temperature. Our goal is to understand the impact of BC addition on the mechanical properties and thermal stability of GBFS and FA geopolymer mortar. Additionally, we assessed the adsorption capabilities of these environmentally friendly geopolymer mortar mixes toward crystal violet dye. This aspect serves as an additional environmental application, considering the limited research on the use of mortar as an adsorbent.

## Materials and methods

### Raw materials

**Figure. 1:**
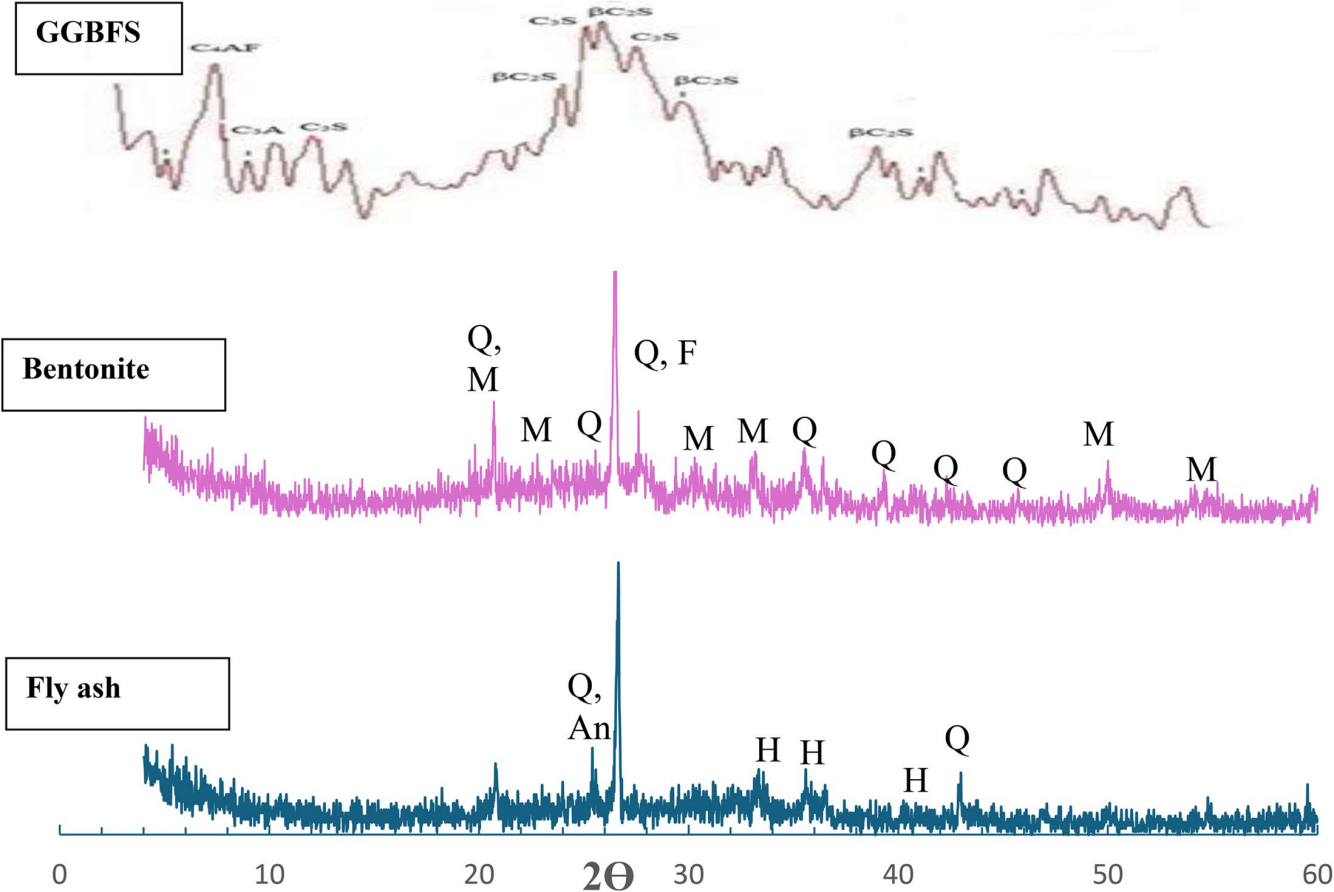
XRD pattern for our study raw materials. (Q = quartz, F = feldspar, M = montmorillonite, An = anatase, H = hematite)

The Helwan Company of Egyptian Iron & Steel supplies GGBFS used in this study. The chemical oxide composition of slag is listed in Table 1, and it has a Blaine surface area of 4700 × 50 cm^2^ per gram. In Egypt, El Nile International Chemical Company in Cairo provides Class C fly ash (FA), while Silica Egypt Company in Burg Al-Arab, Alexandria, Egypt, provides liquid sodium silicate (LSS) with a silica modulus of 2.80 SiO_2_/Na_2_O, which was used in his study. The bentonite clay with a low calcium content was extracted from its natural sources in Egypt. The process involves heating the BC to 800 °C at a rate of 5 °C per minute for two hours. It is then cooled, ground, and sieved until it reaches a size of less than 200 mesh. EL-Goumhouria Chemical Company in Cairo, Egypt, supplies 99% pure NaOH flakes. The supplier of crystal violet (purity 99%, y max = 590 nm) is Prolabo company, France. The chemical oxide content of the raw materials used in this study is listed in Table 1. However, the XRD patterns of FA, GGBFS, and BC are presented in Fig. 1a, b, and c, respectively.

**Table 1:** Chemical composition of raw materials (wt.%).

Material	Oxides											
	SiO_2_	Fe_2_O_3_	Al_2_O_3_	Na_2_O	CaO	MgO	SO_3_	K_2_O	Cl^−^	H_2_O	L.O.I.	TiO_2_
GGBFS	33.71	1.32	17.42	-----	37.52	6.06	1.54	----	0.01	----	< 0.1	----
FA	38.16	14.90	17.20	1.18	18.10	4.76	1.71	1.84	0.06	-----	0.024	1.09
BT	48.52	18.81	9.68	0.14	17.04	3.97	0.07	1.77	----	-----	-----	----
SSL	32.8	-----	-----	11.7	----	-----	----	----	----	55.5	-----	----

### Preparation of geopolymer mortar

To study the effect of bentonite clay on the mechanical properties of geopolymer mortar bars, five mixes were prepared. The mixes included fly ash and slag and had varying amounts of BC (0%, 6%, 8%, 10%, and 12% by weight) in place of FA and GGBFS. Once the mixing process was complete, a flexural strength test was conducted to determine the mechanical properties of the mixes.

#### Alkaline activation solution

The alkaline activation solution was created by mixing the sodium hydroxide (NaOH) and sodium silicate (Na_2_SiO_3_) solutions in a 1:2.5 ratio. This solution was kept as 40% of the total binder quantity. 98–99% pure sodium hydroxide (NaOH) pellets (with a specific gravity of 2.10) were combined with water to create a solution of sodium hydroxide with a molarity of 12 M. Since heat develops after an exothermic reaction, it was prepared 24 h before application.

#### Mixing and curing process

**Figure. 2:**
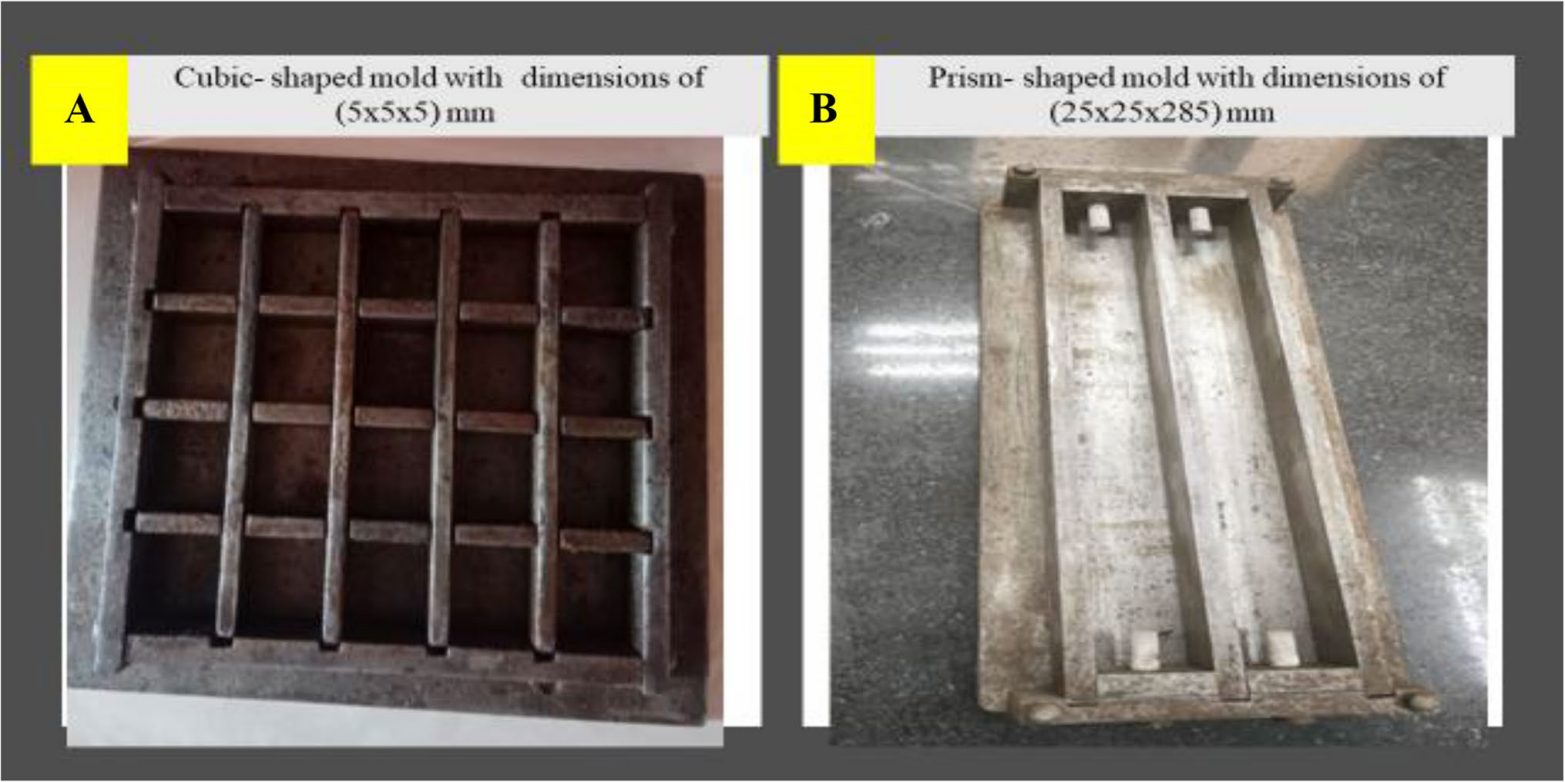
Images of various molds used in our study for (**A**) thermal resistance testing and (**B**) drying shrinkage and flexural strength testing.

To make the geopolymer binder, fly ash, and slag were used as raw materials. To assess the impact of BC, FA, and GBFS were replaced with bentonite at varying percentages (0%, 6%, 8%, 10%, and 12% by weight), and the ratios of each mixture are listed in Table 2. To prevent the mixes from becoming unworkable due to increasing levels of bentonite in the mixtures, a superplasticizer was added. The standard mixing technique was used to make all the mixes, with the mixing process and duration being kept constant for each mix. The mixer was initiated by adding the binder components (FA, GBFS, and BC) after the sand. After three minutes of dry mixing, the prepared alkaline solution was added to the pre-mixed dry ingredients, and superplasticizers were added to ensure homogeneity. The freshly mixed geopolymer mortar bar was poured into molds with dimensions of (25 × 25 × 285) mm (Fig. 2) for the specimens. A needle-type vibrator was used to compact the mixture. To prevent moisture loss from evaporation during the process, the specimens were covered with plastic sheets and kept at room temperature for 48 h. The samples were then removed from the molds and placed in room-temperature water for immersion.

**Table 2:** Mix composition and liquid/solid (L/S) ratio of the examined ternary geopolymer mortar mixes.

Mix ID	Sand (g)	Fly ash (g)	Slag (g)	Bentonite (g)	NaOH (g/cm^3^ )	Na_2_SiO_3_ (g/cm^3^)	L/S ratio	Superplasticizer (SP) (g/cm^3^ )
**SF**	960	320	320	---	183	457.5	0.304	3.18
**SFB**_**6**_	960	300.8	300.8	38.4	183	457.5	0.304	4.1
**SFB**_**8**_	960	294.4	294.4	51.2	183	457.5	0.304	4.5
**SFB**_**10**_	960	288	288	64	183	457.5	0.304	4.6
**SFB**_**12**_	960	281.6	281.6	76	183	457.5	0.304	4.6

### Characterization experiments

#### The mechanical testes of eco-friendly ternary geopolymer mortar based on slag-fly ash-bentonite

##### Drying shrinkage

To measure the drying shrinkage, mortars were made in a prismatic mold with dimensions of (25 × 25 × 285) mm, as illustrated in Fig. 2. After mixing for 3, 7, 14, and 28 days, the drying shrinkage was measured. The samples were removed from the mold and their linear dimensions were measured in millimeters after 24 h of mixing. The samples were then placed in water at a temperature of 23 ± 2 °C. The geopolymer mortars were measured at each age to determine their drying shrinkage.

##### The flexural strength

Mortars’ flexural strength was tested in compliance with NBR 13,279^30^, using a Solo Test machine at a rate of 50 N/s. Prismatic samples, measuring (25 × 25 × 285)mm, were cast and removed from the mold after 24 h. Then, the samples were cured in water at room temperature for 3, 7, 14, and 28 days. The mean value of three samples was used to calculate the flexural strength.

#### The microstructure and morphology characterization of eco-friendly ternary geopolymer mortar samples

Scanning electron microscopy (SEM), Fourier transform infrared spectroscopy (FTIR), and X-ray diffraction (XRD) were employed as experimental methods to investigate the microstructure and morphological characterization of produced eco-friendly ternary geopolymer mortar samples.

- The Bruker D8 Discover diffractometer was used to examine the phase composition of hydration products in geopolymer mortar samples through X-ray diffraction analysis. The use of a Ni-filtered diffractometer in the 2θ range of 4–60°, using Cu-Kα radiation with a voltage of 40 kV and current intensity of 40 mA, provided highly accurate results that revealed crucial information about the samples’ structure, properties, crystallinity, and amorphousness.
- Wave numbers between 400 and 4000 cm^-1^ were selected for the FTIR measurements using a Perkin Elmer 1430 infrared spectrophotometer (USA) using pellets of potassium bromide (KBr).
- The microstructure of the samples was analyzed using an FEI Inspect S 50 scanning electron microscope (FEI Company, Holland), prepared with an energy-dispersive X-ray analyzer (EDAX). The microscope had a power zoom magnification of 300000xs, and an accelerating voltage range of 200 V to 30 kV was used to examine the microstructure of broken composites.

#### Thermal stability test

The 5 × 5 × 5 mm cubic samples (Fig. 2) were subjected to temperatures of 300, 600, and 900 °C to evaluate the thermal stability of eco-friendly geopolymer mortars. At a rate of 5 °C per minute, the 28-day samples were heated to the desired temperatures. After reaching the desired temperature, the furnace was kept up for sixty minutes and then turned off to allow the samples to cool to room temperature. The effectiveness of the mortars with varying compositions was evaluated based on their retained strength (Eq. 1) and percentage of strength loss (Eq. 2) at 300, 600, and 900 ◦C, respectively^31^.

1 $$\mathrm{Retained}\;\mathrm{strength}\;\mathrm{ratio}=\frac{\mathrm{Residual}\;\mathrm{strength}\;\mathrm{at}\;\mathrm{different}\;\mathrm{temperature}}{\mathrm{Initial}\;\mathrm{strength}\;\mathrm{at}\;\mathrm{room}\;\mathrm{temperature}}$$

2 $$Strength\;loss\;\%=\left(1-Retained\;strength\right)\ast100$$

#### Adsorption test

To serve as a stock solution for later studies, a 1000 mg/L crystal violet (CV) solution was prepared and subsequently diluted. In batch mode, 0.05 g of geopolymer (crushed cubes after 7 days of hydration) was added to 20 mL of a 30 mg/L CV dye solution, and the mixture was shaken using a thermostatic orbital shaker. Variables such as pH (2 − 8), adsorbent dosage (0.02–0.15 g), contact time (30–180 min), and starting dye concentration (15–120 mg /L) were examined in a series of batch adsorption tests. The remaining dye concentrations were measured at 590 nm using a UV-vis spectrophotometer. The formula for calculating the dye removal % is as follows^32^:

3 $$\mathrm{Removal}\;\mathrm{percentage}\;\left(\%\right)=\left({\mathrm C}_{\mathrm o}-{\mathrm C}_{\mathrm t}\right)/{\mathrm C}_{\mathrm o}\times100$$

Where the liquid dye concentrations at the beginning and at any time (t) are represented by C_o_ and C_t_ (mg/L), respectively. qe (mg/g), the equilibrium dye adsorption capacity, was calculated using Eq. 4:

4 $$\mathrm{qe}=\left({\mathrm C}_{\mathrm o}-{\mathrm C}_{\mathrm e}\right)\mathrm V/\mathrm W$$

The dye concentrations at starting and equilibrium are denoted by C_o_ (mg/L) and Ce (mg/L), respectively. W (g) is the mass of the dry adsorbent used, and V (L) is the total volume of the solution.

## Results and discussion

### Mechanical properties of eco-friendly ternary geopolymer mortar based on Slag-Fly Ash-Bentonite (SFB)

#### The flexural strength

**Figure. 3:**
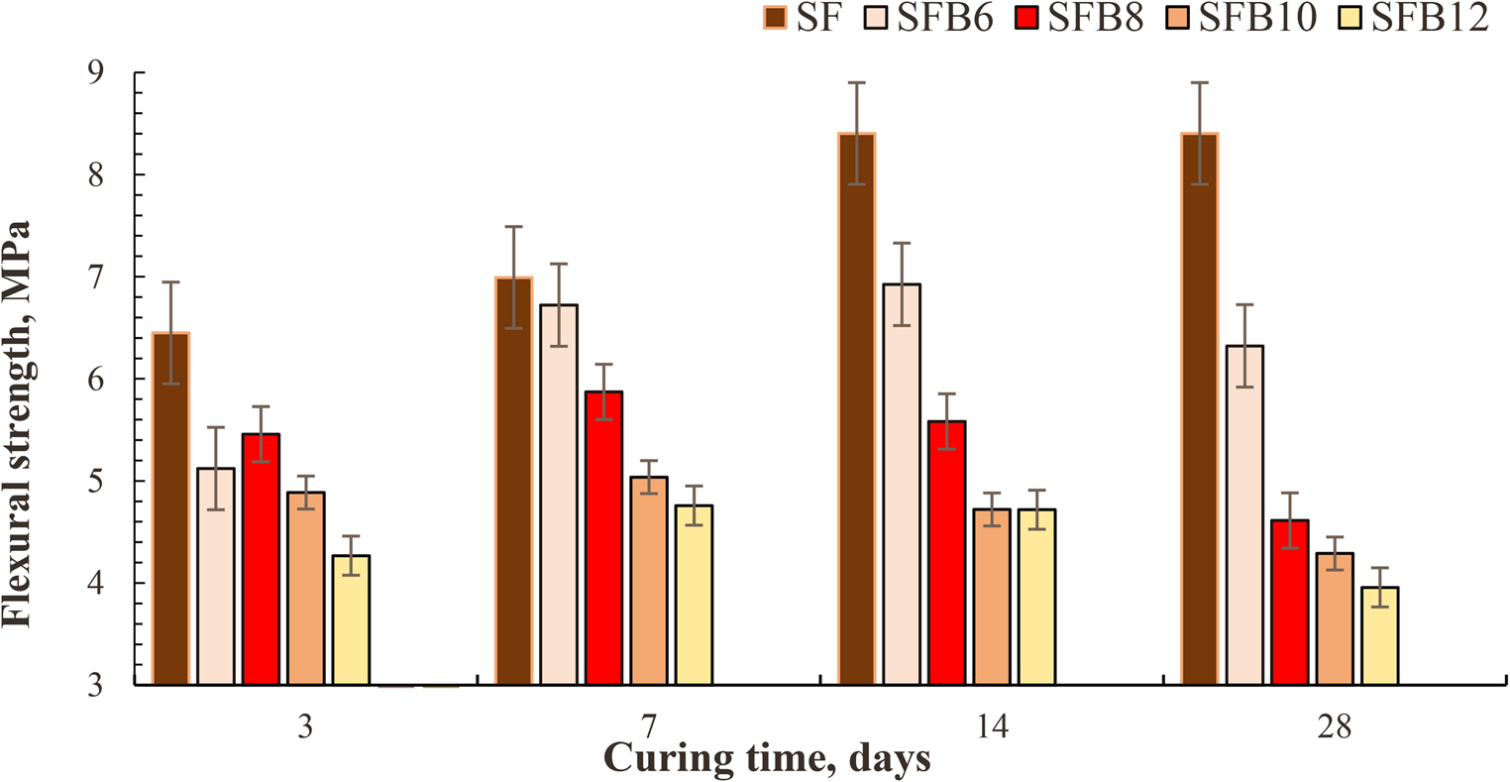
The flexural strength values of SF, SFB 6 , SFB 8 , SFB 10, and SFB 12 , geopolymer mortar after curing at various periods until 28 days.

The flexural strength is a crucial property of mortar bars that determines the load-carrying capacity of structural elements. In this study, it was evaluated the flexural strength of geopolymer mixes with varying replacement levels of bentonite (0, 6%, 8%, 10%, and 12%) at 3, 7, 14, and 28 curing days. The flexural strength of geopolymer mortar mixes (SF, SFB_6_, SFB_8_, SFB_10_, SFB_12_) primarily depends on the replacement level of BC as shown in Fig. 3. Three identical specimens of size (25 × 25 × 285) mm were tested for each mix, and the average strength of the specimens was reported in Fig. 3. The results indicate that the flexural strength of the mix with 6% BC replacement was higher than the mixes with 8%, 10%, and 12% BC replacement. For instance, at 28-day, the flexural strength of the 6% mix increased by 27%, 32%, and 37.5%, respectively, when compared to the same specimens containing 8%, 10%, and 12% BC (Fig. 3). Adding different amounts of bentonite to the mixes causes a drop in flexure strength at all ages compared to the control mix. This decrease in flexure strength is likely due to slower pozzolanic reaction in the early stages, which results in the slower development of calcium silicate hydrate (C-S-H) and calcium aluminate silicate hydrate (CASH) gel^33^. The addition of BC increases the total specific surface, which in turn increases the amount of water needed for mixing and causes a significant decrease in strength values. It has been observed (Fig. 3) that the rate of strength development for the control mix is higher for all hydration ages^33^.

#### Drying shrinkage

**Figure. 4:**
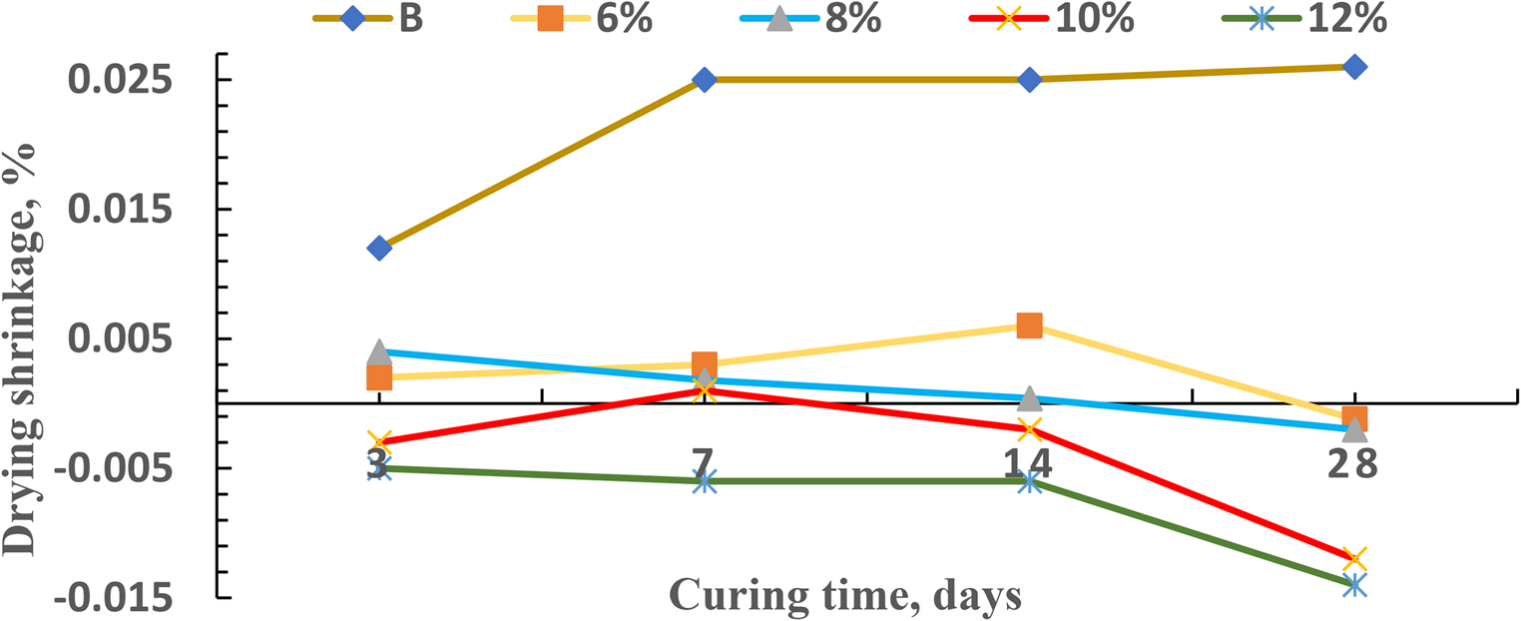
Drying shrinkage values of SF, SFB 6 , SFB 8 , SFB 10, and SFB 12 , geopolymer mortar after curing at various periods until 28 days.

During the process of drying, water evaporation leads to a reduction in volume, which is known as drying shrinkage. After one day of curing, geopolymer mortars were subjected to reduced relative humidity (from 77 to 50% RH), which led to the development of a meniscus in the pore structure and capillary tensions. It is known that water is less chemically absorbed in the polymer matrix, and more water remains free for evaporation during the alkaline reaction, which increases mass loss and influences shrinkage^34^. The drying shrinkage can be influenced by three factors, including pore structure, matrix stiffness, and reaction rate. In contrast to geopolymer, higher energy release due to Portland cement hydration is causing this behavior^35^. Early formation of a denser microstructure reduces water evaporation from capillary holes, and the reduced mass loss is strongly associated with a reduction in drying shrinkage. However, continuous mass loss at later ages does not appear to affect drying shrinkage. Furthermore, the decreased drying shrinkage at early ages is related to the better bond strength (flexural strength) of the geopolymer^36^. The average drying shrinkage of the five specimens is shown in Fig. 4.

### Microstructure Characterization for Eco-friendly SFB ternary geopolymer mortar

#### Characterizing the crystalline phases (XRD)

**Figure. 5:**
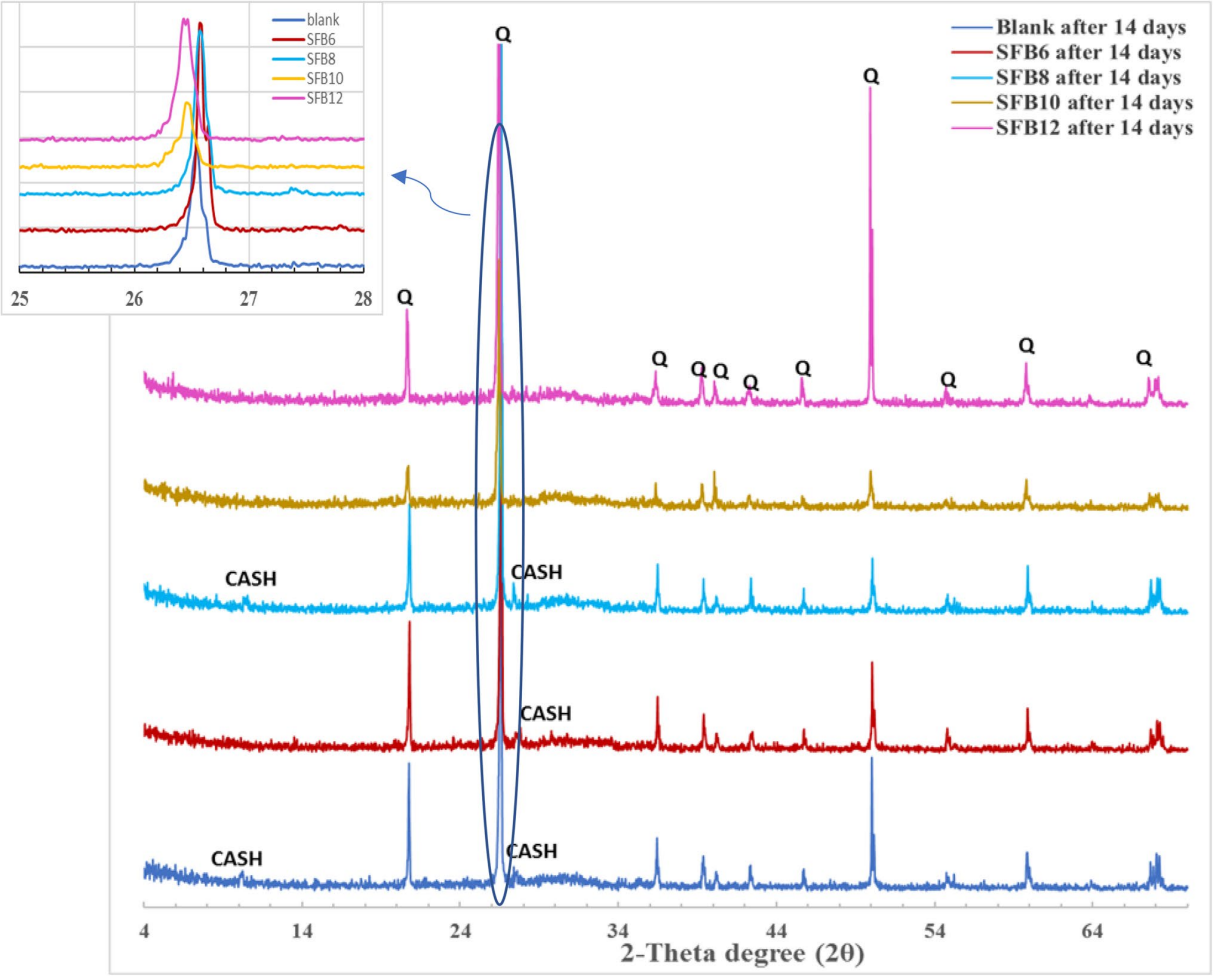
X-ray diffraction patterns of different ternary geopolymer mortar mixes with and without bentonite after 14 days of hydration.

The XRD patterns of various geopolymer mortar mixes, namely SF, SFB_6_, SFB_8_, SFB_10_, and SFB_12_, both with and without BC, are depicted in Fig. 5. The samples were cured for 14 days in water at room temperature. The XRD patterns show the presence of Quartz (Q) (d = 4.25 and 3.34 A^o^) and semi-crystalline aluminosilicate gel. Figure 5 shows that the presence of sand particles in the geopolymer mortar matrix intensifies the diffraction lines related to the presence of quartz, more than in raw materials (Fig. 1). This indicates that the inorganic polymeric components dissolved the parent materials as well, as suggested by references^37,38^. When the amount of BC in geopolymer mortar is increased from 0 to 10% by weight, the intensity of quartz peaks decreases (as shown in Fig. 5). This decrease may be attributed to the negative effect of the increased amount of BC on the geopolymerization process in ternary geopolymer mortar mixes (SFB_6_, SFB_8_, and SFB_10_). However, increasing the BC replacement to 12% by weight increases the intensity of the quartz peak due to the increase in the amount of clay-bentonite in geopolymer mortar (Fig. 5). Although there is a slight shift to a lower angle for the dominant quartz peak from 2θ = 26.61º in the case of the blank sample (SF) to 26.45° in SFB_12_ (Fig. 5), which is another indication of the retardation effect of the bentonite clay on the polymerization process ^39^. According to the mechanical characteristics and XRD results, the ternary geopolymer mortar mix formed by replacing 6 wt% of the blank geopolymer mortar (SF) with bentonite exhibited the best characteristics.

#### Fourier Transform Infrared Spectroscopy (FT-IR)

**Figure. 6:**
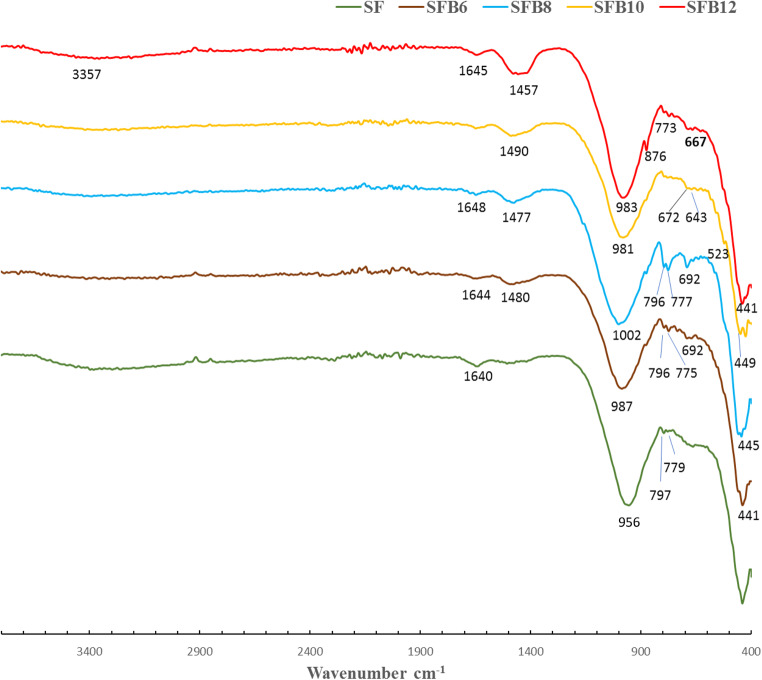
FTIR spectra of different ternary geopolymer mortar mixes with and without bentonite after 14 days of hydration.

After a 14-day hydration period, we examined several mortar samples - SF, SFB_6_, SFB_8_, SFB_10_, and SFB_12_ - and analyzed their FTIR spectra, which are shown in Fig. 6. The stretching O-H vibrations and the bending H-O-H vibrations of the water molecules inside the geopolymer network are responsible for the bands observed at wave numbers 3357–3382 cm^−1^and1640–1648 cm^−1^, respectively^40,41^. The most significant peak in the FTIR spectrum falls in the range of 956–1002 cm^−1^ (Fig. 6), and it corresponds to the asymmetric vibrations of Si-O-T (T = Si, Al) bonds in aluminosilicate minerals^40^. In the SFB_8_ spectrum, this peak shifted to a higher wavenumber of 1002 cm^−1^, indicating a slowdown in the geopolymerization process (Fig. 6). However, in the SF spectra, the same peak shifted to lower wavenumbers of approximately 956 cm^−1^ than other mortar mixes. This shift is attributed to an increase in the Al^3+^ substitution of Si^4+^ in the silicate framework, which leads to the formation of additional Al-enriched geopolymer phases, ultimately accelerating the geopolymerization process^40,41^. The intensity of the bands in the range of 779 –773 cm^−1^ indicates the bending vibrations of (Si-O-Si or Al-O-Si), which belong to Al in tetrahedral positions, while the weak peak at 523 cm^−1^ corresponds to bending vibrations of Si–O–Al^VI^ for the Al in octahedral positions respectively^40,41^. These vibrations are an indication of the geopolymerization reaction. In addition, the peak of strong alumina at 797 cm^−1^, the absorption band belonging to the bending Al-O in AlO_6_ octahedral coordination, decreased in intensity in the case of the SF mix, as shown in Fig. 6. This suggests that the geopolymerization reaction took place ^42^. For all geopolymer mortars that had BC (SFB_6_, SFB8, SFB_10_, and SFB_12_), the asymmetric vibration peak of ν Si-O-T shifted to a higher wavenumber, while the intensity of the alumina peak at 793 cm^−1^ increased compared to the SF mix. This increase is attributed to the addition of bentonite, which inhibits the geopolymerization process. Additionally, the intensity of the asymmetric vibrations of the O-C-O bonds of sodium carbonate at a wavenumber of about 1401–1454 cm^−1^ increased with the increasing amount of BC as shown in Fig. 6. This increase is another indication of a reduction in the formation of hydration products, which fill the open pores and prevent the carbonation process. Finally, the quartz in the mortar samples’ fine aggregate is responsible for the bands at wave numbers 690–692 cm^−1^ and 449–441 cm^−1^^40^.

#### Scanning electron microscopy

**Figure. 7:**
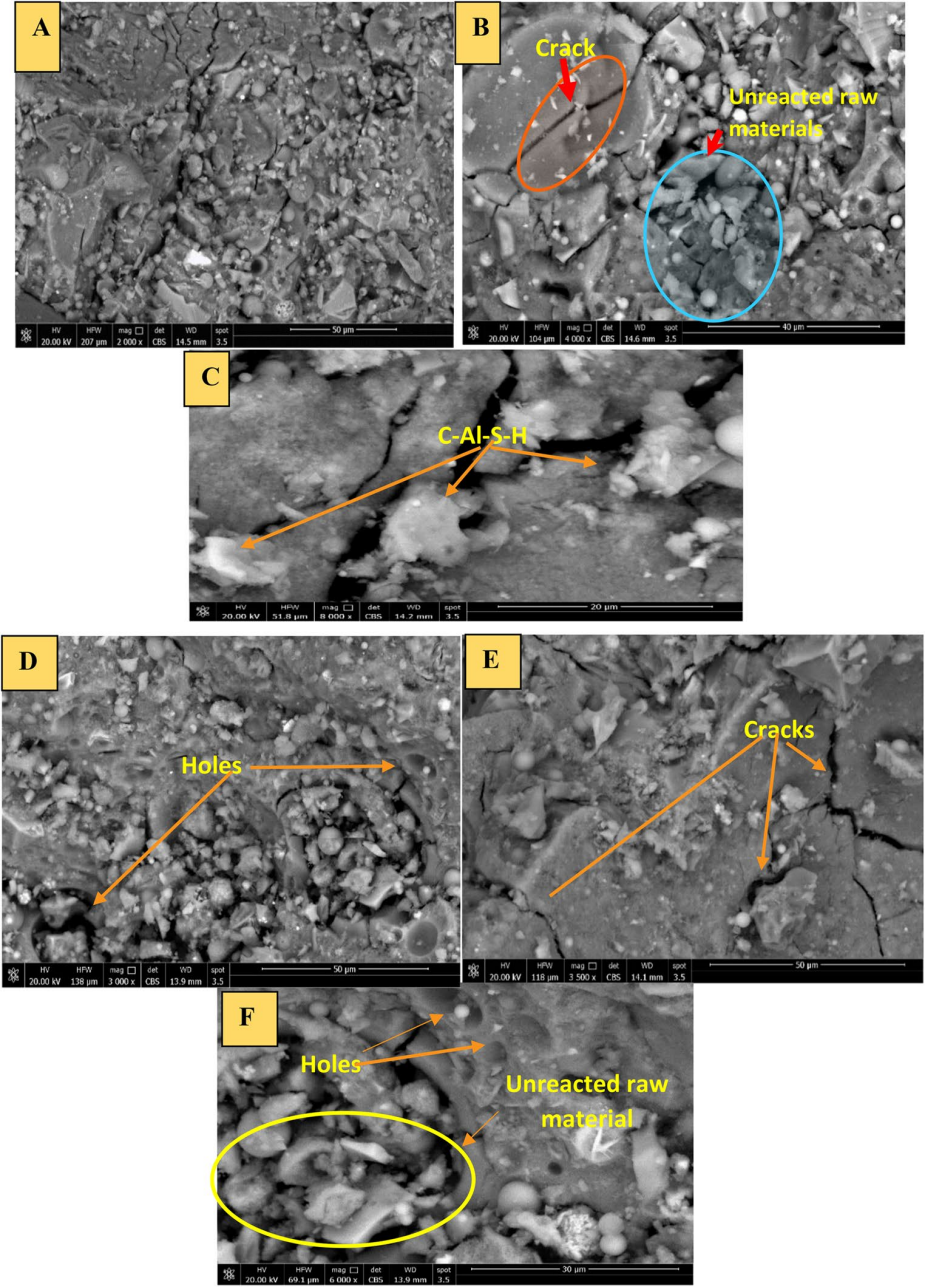
SEM micrograph of some selected geopolymer mortar mixes after 14 days of curing; with different magnifications: (**A**) SFB6 -2000x, (**B**) SFB6 - 4000x, (**C**) SFB6 - 8000x., (**D**) SFB12- 3000x, (**E**) SFB12 -3500x, and (**F**) SFB12 -6000.

Figure 7 summarizes SEM micrographs of some selected geopolymer mortar samples cured under water for 14 days with varying BC ratios (6% and 12%) and different magnifications. The micrographs indicate that the geopolymer mortars, SFB_6_ and SFB_12_, have a dense and uniform microstructure that is mainly composed of aluminosilicate gel due to the geopolymerization process. However, there are small microcracks in the dense matrix caused by a weak interfacial connection between the aggregate particles (sand) and the paste matrix^40^. The microstructure of SFB_6_ (Fig. 7A, B, and C) showed fewer cracks and a denser interfacial transition zone (ITZ) compared to SFB_12_ (Fig. 7D, E, and F). This could be due to the lower amount of BC (6%) and larger amounts of slag and fly ash in the SFB_6_ mixture, which play an important role in improving the geopolymerization process^43^. According to Jindal’s research^43^, geopolymer materials containing slag have a strong bonding strength. As last shown in Fig. 3, sample SFB_6_ had the highest flexural strength among all the bentonite geopolymer mortar samples studied for all hydration ages, which was attributed to its higher gel formation due to the presence of a larger amount of slag. The greater amount of gel formed in the SFB_6_ mixture induced strong cohesion between the binder and sand particles, consequently leading to stronger strength development^40,43^. Figure 7(D, E and F) depicts the microstructure of the geopolymer mortar mix SFB_12_, revealing numerous holes and a significant amount of unreacted raw material particles. Furthermore, it exhibits severe cracks and weak interfacial bonding between the paste matrix and aggregate particles, which aligns with the low flexural strength of SFB_12_ as demonstrated in Fig. 3. This could be attributed to the diminished geopolymerization process resulting from the increased addition of BC, ranging from 6–12%, ultimately leading to a decrease in flexural strength. All of these results are in good agreement with flexural strength, drying shrinkage, XRD, and FTIR results.

### Fire resistance results of prepared eco-friendly geopolymer mortar samples

#### Physical appearance

**Figure. 8:**
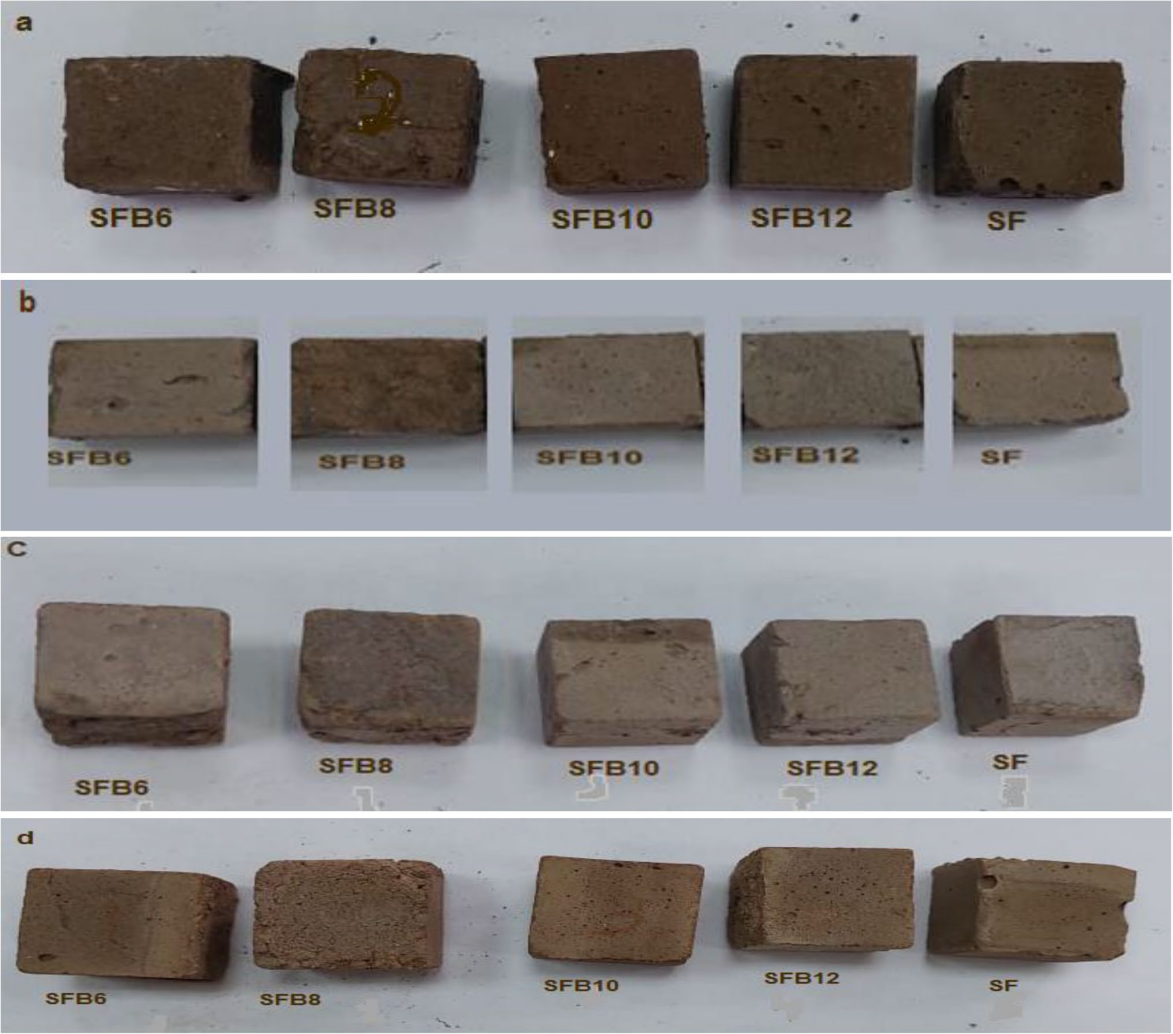
Changes in the appearance of geopolymer mortar at (**a**) room temperature, (**b**) 300 o C, (**c**) 600 o C, and (**d**) 900 o C.

The physical characteristics of geopolymer mortars containing BC at 300, 600, and 900 °C are shown in Fig. 8. Before temperature exposure, the geopolymer specimen’s mortar had a brownish-red color. This changed to a light brown color at 300 °C, and a grey color at 600 °C, due to the moisture loss that took place in the geopolymer matrix (drying). Then, changed to a light reddish-brown at 900 °C (Fig. 8). This happened because various compounds’ elemental iron underwent oxidation to produce red iron oxide^44,45^. The geopolymer mortar specimens were found to have no cracks or pores after being exposed to 300 °C, as shown in Fig. 8. This was due to the sand content in the mixture, which replaced a portion of the geopolymer matrix, reducing the pore pressure and crack formation. Furthermore, aggregates can also restrict the development of cracks; as a result, the sand in the geopolymer may hinder the start and advancement of cracks^46^.

However, after exposure to temperatures of 600 °C and 900 °C, the samples did not display any signs of cracks, but some pores were visible on their surface (as seen in Fig. 8). These pores were caused by the incompatibility between the expansion of sand and the shrinkage of the geopolymer matrix.

#### Compressive strength and strength loss

The retained strength ratio and the percentage of strength loss from Eqs. (1) and (2) were used to physically measure the mortars’ durability under elevated fire conditions and are presented in Table 3. The compressive strengths of the geopolymer mortar specimen before and after heating are also shown in Table (3). For blank geopolymer mortar, the compressive strength first increases up to 600 °C before decreasing above that temperature. The geopolymerization of unreacted precursors led to improvements in compressive strength of 16.7% at 600 °C^47^. Subsequently, the mixes containing BC show the same trend as blank geopolymer mortar till 600. The compressive strength of mortars made with SFB_6_, SFB_8_, SFB_10_, and SFB_12_ improved by 15.0%, 30.0%, 32.8%, and 41.2%, respectively. It was observed that the increase in compressive strength values was slightly higher in mortars produced with BC than in the blank mortar, up to a temperature of 600 °C. This is because some of the raw material particles remained unreacted during the polymerization process (Fig. 7), which started the condensation process during the first heating period, resulting in increased compressive strength^48^. Furthermore, BC can bind sand grains in mortar, creating a plastic paste that helps the mortar mix become workable when water is present^19^. Additionally, all varieties of mortars showed a significant negative value of strength loss, with a maximum value of 42.0% for SFB_12_.

**Table 3:** The compressive strength of geopolymer mortar at elevated temperature.

Mix ID	Compressive Strength (MPa)				Retained strength ratio			Strength loss %		
	initial	300^o^C	600^o^C	900^o^C	300^o^C	600^o^C	900^o^C	300^o^C	600^o^C	900^o^C
SF	25	28	30	20	1.12	1.07	0.66	-12.0	-7.0	34.0
SFB_6_	17	18	20	15	1.05	1.11	0.75	-5.0	-11.0	25.0
SFB_8_	7	8	10	--	1.14	1.25	--	-14.0	-25.0	--
SFB_10_	15	20	22	25	1.33	1.10	1.14	-33.0	-10.0	-14.0
SFB_12_	10	12	17	15	1.20	1.42	0.88	-20.0	-42.0	12.0

The specimens exhibiting the highest percentage of strength loss at 900 °C were the SF and SFB_6_ mortars, with a percentage of 34% and 25%, respectively. Furthermore, the SFB_10_ mortars showed the lowest percentage of strength loss. At 900^o^C, the compressive strength of all mixes decreases except for SFB_10_ (Table 3). This is due to increasing temperature to 900^o^C accelerated the dehydroxylation process, which broke down the geoplymerization product and reduced their compressive strength^49^. In particular, geopolymer mortar produced with 10% BC achieved a 40% increase in compressive strength at this temperature. The compressive strength value of SFB_8_ is unusual and might be the result of faulty equipment or experiments.

#### Microstructure Characterization for SFB- ternary geopolymer mortar after thermal treatment

##### X-ray Diffraction Analysis (XRD)

**Figure. 9:**
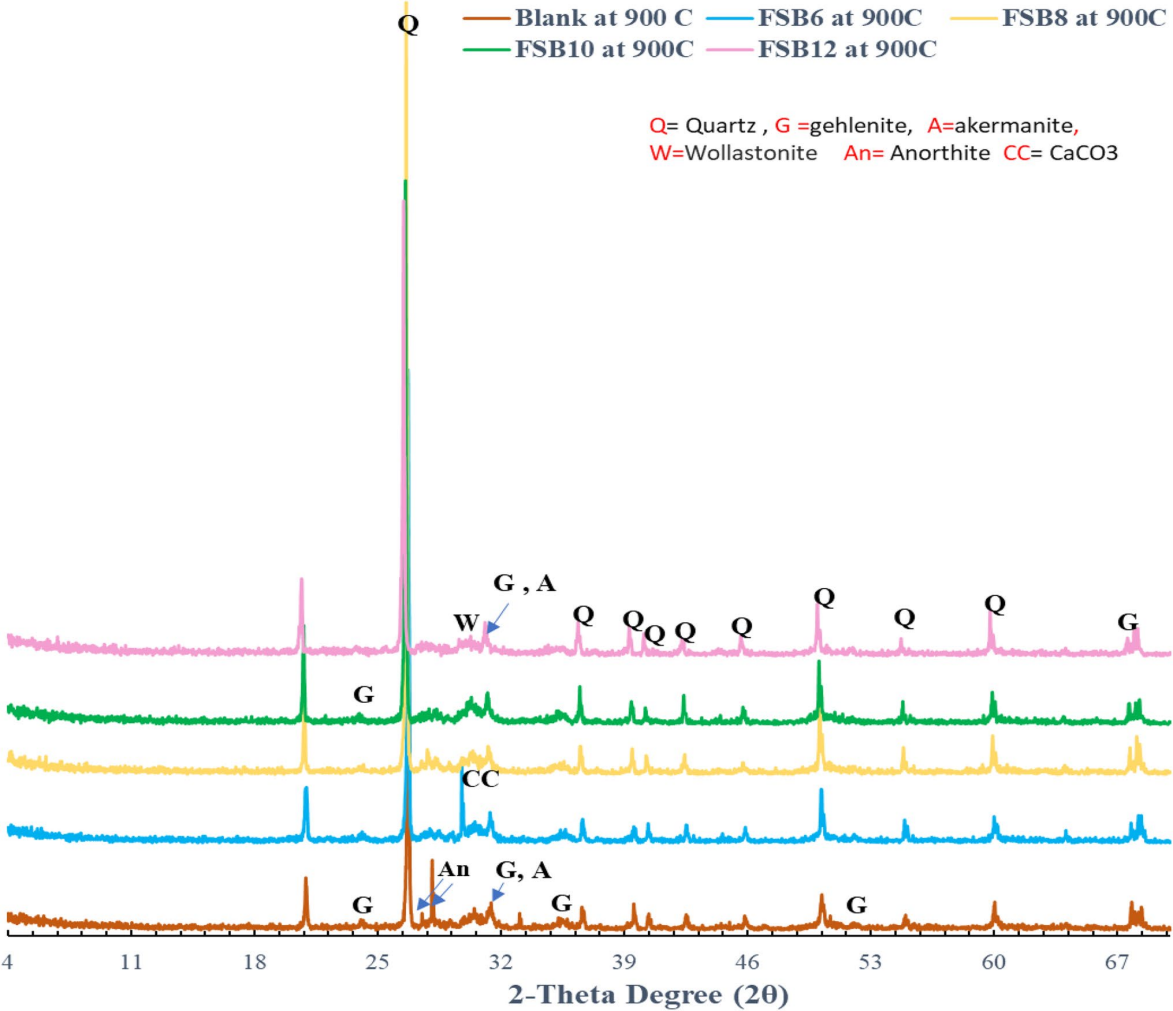
X-ray diffraction patterns of SF (blank) and different SFB-ternary geopolymer mortar mixes after heat treatment to 900ºC.

The XRD results of various eco-friendly geopolymer mortar mixes (SF, SFB_6_, SFB_8_, SFB_10_, and SFB_12_) after being cured in water at room temperature for 28 days and then exposed to 900 °C, are shown in Fig. 9. After subjecting the geopolymer mortar specimens to 900 °C, the XRD patterns underwent significant changes when compared to their room-temperature counterparts (Fig. 5). The peak corresponding to C-A–S–H disappeared, and the amorphous phase of N-A-S-H is associated with the broad hump at 20^◦^–40^◦^ of 2θ observed (Fig. 9). In addition, the peaks of new crystalline anhydrous compounds such as akermanite (Ca_2_MgSiO_7_), gehlenite (Ca_2_Al[AlSiO_7_]), wollastonite (CaSiO_3_), and anorthite (CaAl_2_Si_2_O_8_) was readily apparent (Fig. 9)^50–52^. As a result of N-A-S-H dehydration and breakdown at temperatures above 600 °C the geopolymer strength was reduced^53,54^. A weakened broad hump is observed in all mixes except for SFB_10_ (as seen in Fig. 9) have established the correlation between the reduction in geopolymer strength and N-A-S-H fraction and amorphous content. On the other hand, the sharp peaks observed at 2θ = 26.6^°^ are assigned to quartz (SiO_2_). The quartz peak was extremely prominent because of the quartz sand powder used in the preparation of mortars^52^. It was observed that all geopolymer mortar mixes showed a decrease in the strength of quartz peaks when exposed to 900 °C (Fig. 9), as opposed to the intensity of the same peaks after curing at room temperature (Fig. 5). The primary reason for this was that the link between the geopolymer matrix and quartz sand was broken by the dehydration and breakdown of the matrix as well as thermal incompatibility^52^. On the other hand, the characteristic peaks related to crystalline products and quartz were higher in the case of SFB_10_ geopolymer mortar mix as seen in Fig. 9, resulting in a more compact structure after high-temperature treatment. This is consistent with the compressive strength results.

##### Scanning Electron Microscope (SEM)

**Figure. 10:**
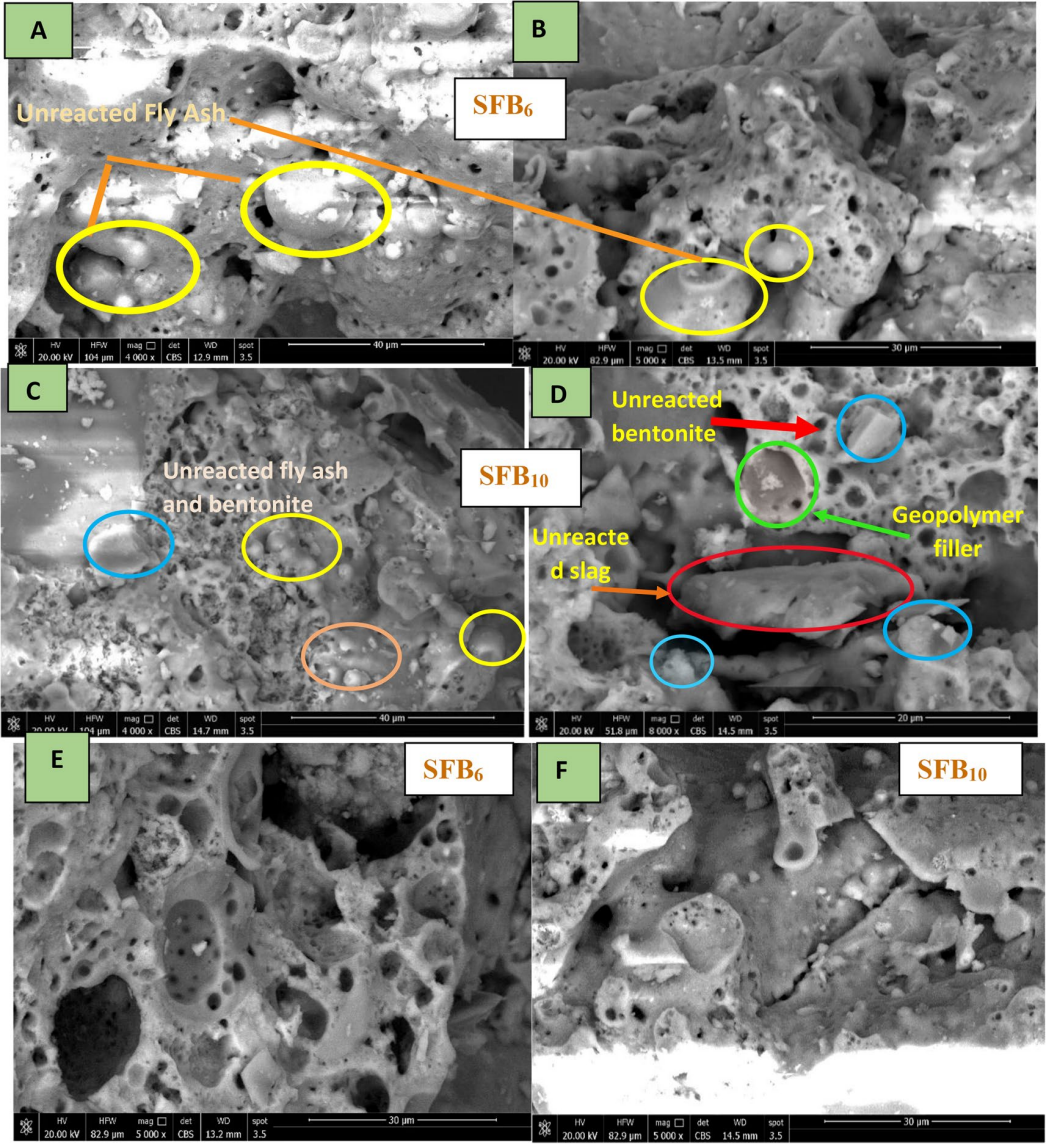
SEM micrograph of some chosen geopolymer mortar mix after heating to 900ºC; with different magnification (**A**) SFB 6 -4000x, (**B**) SFB 6− 5000x, (**C**) SFB 10 - 4000x, (**D**) SFB 10− 8000x, (**E**) ) SFB 6 – 5000X, and (**F**) SFB 10 – 5000x.

The microstructure of some chosen samples of SFB-based geopolymers, with varying bentonite contents (6% and 10%), was analyzed using SEM after exposure to high temperatures. Figure 10 display the results. Heating geopolymer mortars to 900 °C caused the breakdown of hydration products, leading to the formation of large microcracks and holes on the geopolymer mix microstructure ^55^. The SF_6_ sample, when subjected to 900 °C, formed numerous cracks and a higher number of holes in the surface micro-structure, as shown in Fig. 10A and -B. This suggests that the CASH underwent hydrolysis or decomposition, resulting in thermal strains and temperature gradients that caused an increase in damage ^55^. According to the last study by Slany et al. ^50^, bentonite dihydroxylation occurs between 400 and 800 °C. Figure 10A and B shows a small amount of unreacted material, such as FA particles. However, the SFB_10_ sample (Fig. 10C, and D) exhibited a higher proportion of unreacted raw materials after being heated to 900 °C. Furthermore, it had the lowest number of pores (Fig. 10E, and F) and the highest compressive strength of 25 MPa (Table 3). All geopolymer samples exhibited strength gain at 600 °C due to the polymerization of initially unreacted materials, leading to the densification of the matrix and an increase in the T-O-Si band of all geopolymers ^51,56,57^. The decrease in the number of pores in the SFB_10_ sample after heating may be attributed to the increase in the amount of unreacted material in its geopolymer matrix, which begins to react after a temperature of 600 °C^51^. This forms a geopolymer filler that fills the open pores and increases the densification of the matrix at 900 °C ^51^. SFB_6_ has a lower compressive strength 15 MPa after being heated to 900 °C (Table 3) due to the presence of a large number and size of pores in the geopolymer matrices compared to the SFB_10_ mix, as shown in Fig. 10E, and F. In general, increasing the amount of BC to 10% in the geopolymer enhances the thermal stability of the ternary geopolymer mortar matrix (Fig. 9E, and F). These observations are in good agreement with compressive strength, XRD, and FTIR results.

##### Fourier Transform Infrared Spectroscopy (FT-IR)

**Figure. 11:**
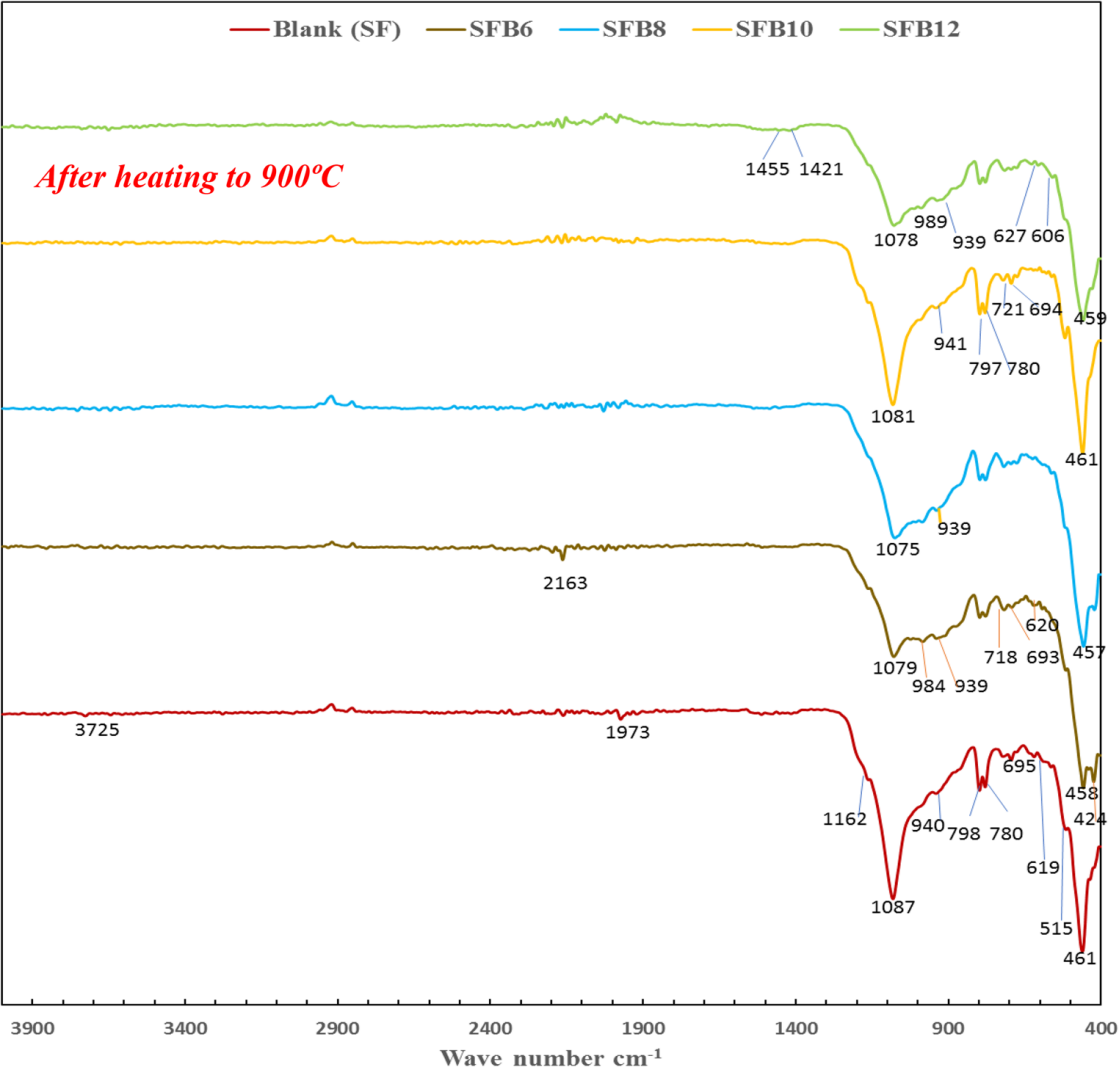
FTIR spectra of different ternary geopolymer mortar mixes with and without bentonite after heating to 900 °C.

The IR spectra confirm that dehydroxylation took place during calcination, as shown in Fig. 11. The absorption bands at 3358 and 1645 cm^−1^ in Fig. 6, which are associated with the stretching and bending vibrations of OH in water, disappeared due to the dehydration and dehydroxylation of hydration components inside geopolymer mixes during calcination at 900 °C. As a result of thermal treatment also, the breakdown of carbonates was observed, which is shown in Fig. 11 by the complete disappearance of the asymmetric vibrations of νO-C-O at 1457–1480 cm^−1^ (Fig. 6). The FTIR spectra of SFB_12_ exhibit the presence of carbonate due to a weak absorption band at 1420 cm^−1^, in addition to the peak at 1455 cm^−1^. The broadening of the prominent band that resulted from the Si-O stretching vibration and the shift of its maximum to higher wavenumbers from ranges of 1002 –956 cm^−1^ (Fig. 6) to 1085 –1075 cm^−1^ (Fig. 11) indicated structure disordering. Additionally, instead of clear and distinct absorptions within the region of νSi-O vibrations (Fig. 6), a broad band between 1162 and 940 cm^−1^ was created (Fig. 11). For all geopolymer mortar mixes, the broadband of the silicate phase has divided into three distinct absorption bands at around 1081 cm^−1^, 984 cm^−1^, and 939 cm^−1^, as seen in Fig. 11. This is attributed to the crystallization process which causes the gel structures of C-S-H and N-A-S-H to disintegrate ^51^. In addition, the XRD findings in Fig. 9 corroborate this indication of the creation of the wollastonite phase. Structural disordering also caused the appearance of a broad range of wave numbers from 600 to 400 cm^−1^^50,51^. The bands observed in different geopolymer mortar mixes (SF, SFB_6_, SFB_8_, SFB_10_, and SFB_12_) at 695–780 and 515–619 cm^−1^ are related to zeolites and are associated with the asymmetric stretching, bending, and rocking vibrations in the double rings of T–O^52^. The intensity of these bands is higher in the case of SF and SFB_10_ mortar mixes than in other mixes because of the strengths that SF and SFB_10_ gained after being exposed to 900 °C (20 and 25 MPa), as shown in Table 3. This gives further evidence of geopolymerization of unreacted material after exposure to high temperatures and the formation of zeolite products which filled open pores and increased the strength^51^. The band observed at 625 cm^−1^ is associated with the bending vibration of Al–O groups, while the one at 423 cm^−1^ is linked to the bending vibrations of O-Al-O^50,52^. Additionally, the characteristic absorption band of silica (α-quartz) can be seen at 797 cm^−1^^50^. In comparison to other mortar mixes, SF and SFB_10_ have a more intense band at 797 cm^−1^, which when combined with the band at 1087 cm^−1^ indicates a greater amount of amorphous silica^50,51^. The most intensive band at wave number around 457–461 cm^−1^ (Fig. 11) of bending vibration O-Si-O which is typical for geopolymer gel appeared as a series of peaks in IR spectra of all geopolymer mortar matrices. The presence of a band at approximately 461 cm^−1^ after calcination is evidence of the formation of amorphous silica and an increased degree of geopolymerization^56,57^.

### Environmental application of different prepared eco-friendly geopolymer mortars as adsorbent

This study explores the effectiveness of eco-friendly geopolymer mortars in removing crystal violet dye from wastewater. The adsorption capacity of these mortars provides valuable insights into their potential for water treatment, contributing to the development of sustainable and efficient wastewater treatment methods. Various factors influencing the adsorption behavior of eco-friendly geopolymer mortars, including pH, adsorbent dose, time, and dye concentration, were examined.

#### Effect of pH

**Figure. 12:**
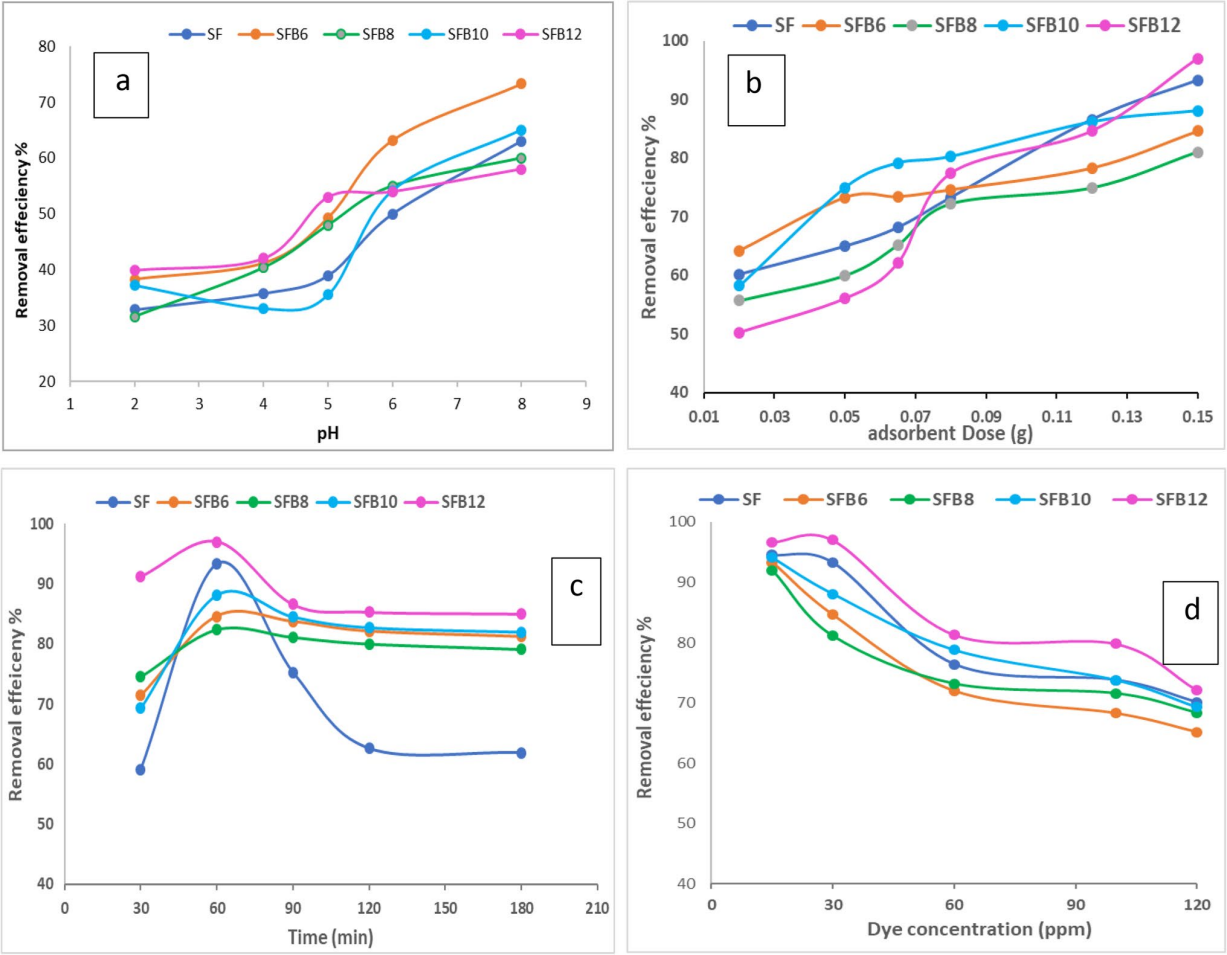
The effect of (**a**) solution pH, (**b**) adsorbent dose, (**c**) contact time, and (**d**) initial concentration of the crystal violet dye, on the dye removal efficiency using different geopolymer mortar mixes.

The degree of ionization, speciation, and surface charge of the geopolymer can all be impacted by pH, making it a critical factor that could affect the adsorption process. So, as shown in Fig. (12a), the influence of pH on bentonite geopolymer-assisted CV removal is investigated at pH values of 2, 4, 5, 6, and 8. Because of its alkaline properties, the adsorbent surface is more favorable for the adsorption of cationic species, including CV dye, because of their negative charge^58^. At a strongly acidic medium, due to electrostatic forces that repel positively charged geopolymer sites, the protonation of geopolymer functional groups significantly decreased CV removal^59^. The elimination efficiency rises as the solution’s pH increases. A pH = 8 solution had the maximum removal effectiveness. Furthermore, at pH = 8, cationic dye adsorption was more favorable because a higher electrostatic attraction force could be formed between the positively charged crystal violet group and the functional group that has a negative charge on the adsorbent surface^58^. Additionally, one of the main factors contributing to the increased dye removal was the hydrogen interaction between the CV dye molecule and the –OH groups of the geopolymer surface^59^. All of the CV adsorption tests were conducted at pH 8.0.

#### Effect of dosage

The results of the investigation into the impact of dosage on the elimination of CV using geopolymer are illustrated in Fig. (12b). As a result, the elimination efficiency is increased when the dosage is increased from 0.02 to 0.15 g. This results from an increase in active sites at higher dosages^60,61^. The maximum removal efficiency (97%) is observed for the SFB_12_ mix. Accordingly, the experiment could proceed with the 0.15 g dose.

#### Effect of time

In this study, we examined how the duration of contact time affects the adsorption of CV by the geopolymer adsorbent. Figure (12c) demonstrates that increasing the contact time from 30 to 60 min improves the removal efficiency by up to 97%. However, when the contact time is extended to 180 min, there is a slight decrease in removal efficiency. We observed that a significant amount of dye is absorbed within the first 30 min, and the adsorbent reaches equilibrium at 60 min. This rapid adsorption is due to the presence of available adsorption sites at the beginning of the process^62–64^. Once these sites are saturated and equilibrium is reached, further adsorption does not occur^62–64^.

#### Effect of dye concentration

The effect of starting CV on the whole geopolymer adsorbent performance at equilibrium is assessed within the range of 15–120 mg/L. As displayed in Fig. (12d), the removal efficiency is decreased when the concentration of CV is increased from 15 to 120 mg/L. This is a result of active adsorption sites becoming saturated due to an increase in CV concentration^65^.

Applying the Langmuir and Freundlich models, we were able to optimize the design of an adsorption system that would remove the crystal violet dye from the surface of several eco-friendly geopolymer mortar mixes (SF, SFB_6_, SFB_8_, SFB_10_, and SFB_12_). The adsorbent surface’s homogeneity and the idea that adsorption takes place on a monolayer underpin the Langmuir model. Conversely, the Freundlich model is predicated on the notion that adsorption takes place on a heterogeneous surface and that it is possible for several layers to develop^32,66^. The maximum adsorption capacity of our selected adsorbent and its variation with concentration and temperature were ascertained by employing both models^32,66^.

Equation (5) of the Langmuir Model was applied to maintain the dye’s equilibrium isotherms on various geopolymer mortar adsorbents^66^.

5 $$Ce/qe=1/q_{max}K_L+Ce/q_{max}$$

**Figure. 13:**
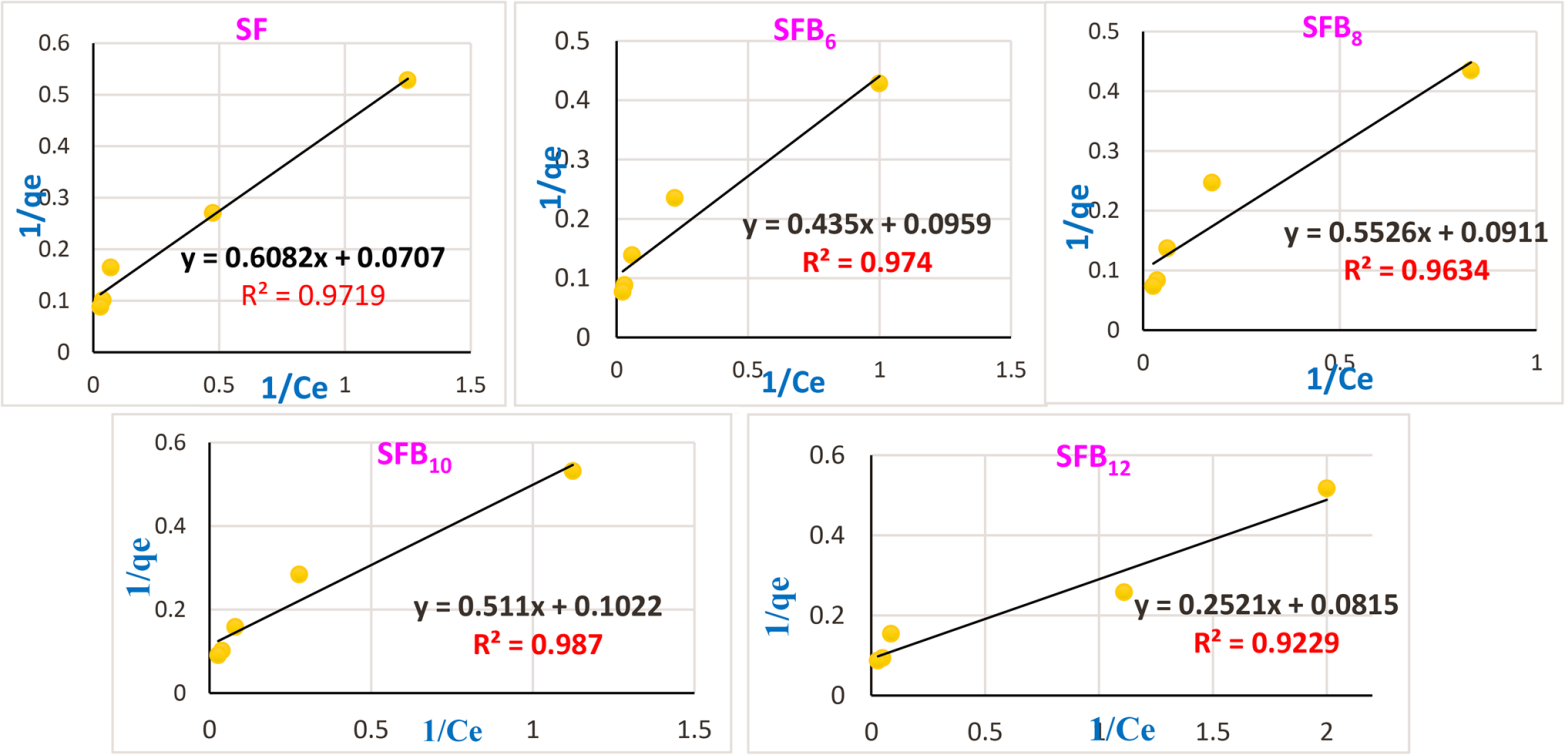
Langmuir model for the adsorption process onto the surface of different geopolymer mortar mixes toward crystal violet dye.

Ce is the equilibrium concentration of adsorbate, qe is the adsorbed dye per unit mass at equilibrium (mg/g), K_L_ is the Langmuir Isotherm constant (L/mg), and q_max_ is the maximum capacity of adsorbent for adsorbate (mg/g). The plot of 1/qe versus 1/Ce for different geopolymer mortar mixes (SF, SFB_6_, SFB_8_, SFB_10_, SFB_12_) depicted in Fig. 13 yields a linear relationship with an intercept equal to 1/qmax and a slope of 1/qmax. KL. Equation 6 below defines a separation factor (R_L_), crucial for characterizing key elements of the Langmuir isotherms^66^:

6 $$R_L=1/\left(1+R_LC_0\right)$$

On the contrary, the following is the Freundlich equation^32,66^:

7 $$\mathrm{Log}\;\mathrm{qe}=\log\;{\mathrm K}_{\mathrm F}+1/\mathrm n\;\log\;\mathrm{Ce}$$

**Figure. 14:**
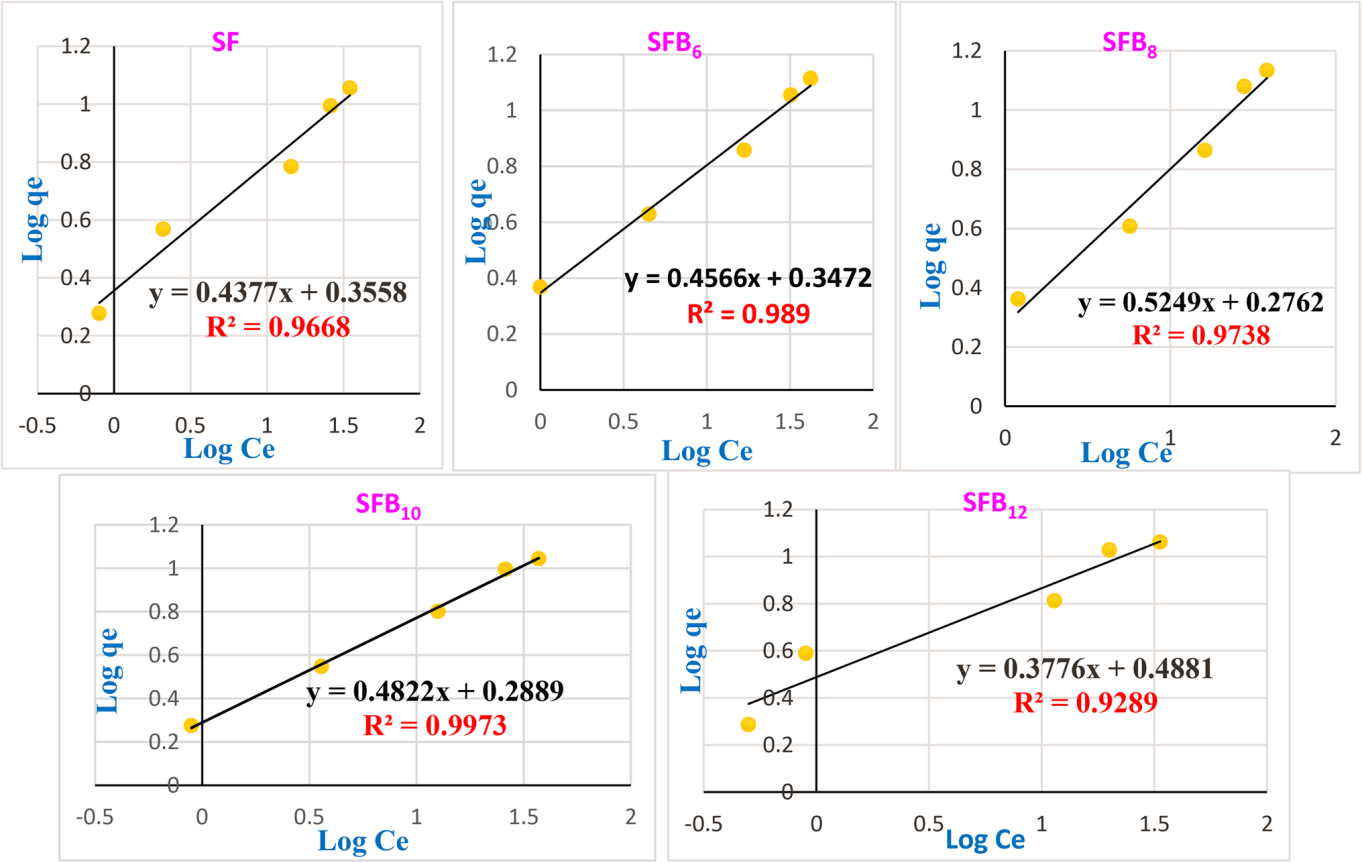
Freundlich model for the adsorption process onto the surface of different geopolymer mortar mixes toward crystal violet dye.

The adsorption capacity is determined by the intercept K_F_ (mg g^−1^)(L mg^−1^)^1/n^ derived from the graph of log qe versus log Ce (Fig. 14). The slope (1/n) which represents the adsorption intensity or surface heterogeneity, indicates the favorability and capacity of the adsorbent–adsorbate system^32,66^. Table 4 indicates that the n values for different geopolymer mixes ranged from 1.90513 to 2.64831, falling between 1 and 10. Additionally, K_F_ values for various geopolymer mortar mixes ranged from 1.889 to 3.077 (mg g^−1^) (L mg^−1^)^1/n^, indicating that KF > 1. The K_L_, Q_max_, and R_L_ values for crystal violet dye in different geopolymer mortar mixes are also presented in Table 4. The Q_max_ values for the various geopolymer mortar mixes varied from 9.78 to 14.14 mg/g, suggesting that the adsorption activity of mortar mix SF is greater than that of other geopolymer mortar mixes. The values of (R^2^) show that the adsorption process was favorable, as they lie in the range of zero to one (for both isotherm models), as demonstrated in Figs. (13, and 14) and Table 4. All geopolymer mortar mixes, namely SF, SFB_6_, SFB_8_, SFB_10_, and SFB_12_, have positive K_L_ values smaller than unity (Table 4), suggesting an enhanced sorption affinity^32,66^. Furthermore, all geopolymer mortar mixes have an R_L_ value of less than 1, supporting favorable adsorption^66^. The isotherm results demonstrated that the adsorption interaction process between different geopolymer mortar mixes and crystal violet dye was favorable and in line with the Freundlich theory, as shown in Figs. 13 and 14; Table 4. This suggests that the adsorption process for CV dye was mainly multilayer on heterogeneous sites. Therefore, geopolymer mortar mixes (SF, SFB_6_, SFB_8_, SFB_10_, and SFB_12_) show promise for water treatment applications by effectively eliminating crystal violet dye from wastewater.

**Table 4:** Isotherm measurements for different geopolymer mortar adsorbents.

Adsorbent	Langmuir Isotherm				Freundlich Isotherm			
	Q_max_ (mg/g)	K_L_ (L/g)	*R*_L_	*R* ^2^	1/*n*	*n*	KF (mg g^−1^ )(L mg^−1^ ) ^1/*n*^	*R* ^2^
SF	14.14	0.116	0.2229	0.972	0.438	2.28467	2.269	0.967
**SF**_**6**_	**10.43**	**0.221**	**0.1313**	**0.974**	**0.457**	**2.19010**	**2.224**	**0.989**
**SF**_**8**_	**10.98**	**0.165**	**0.1682**	**0.963**	**0.525**	**1.90513**	**1.889**	**0.974**
**SF**_**10**_	**9.78**	**0.200**	**0.1429**	**0.987**	**0.482**	**2.07383**	**1.945**	**0.997**
**SF**_**12**_	**12.27**	**0.323**	**0.0935**	**0.923**	**0.378**	**2.64831**	**3.077**	**0.929**

**Figure. 15:**
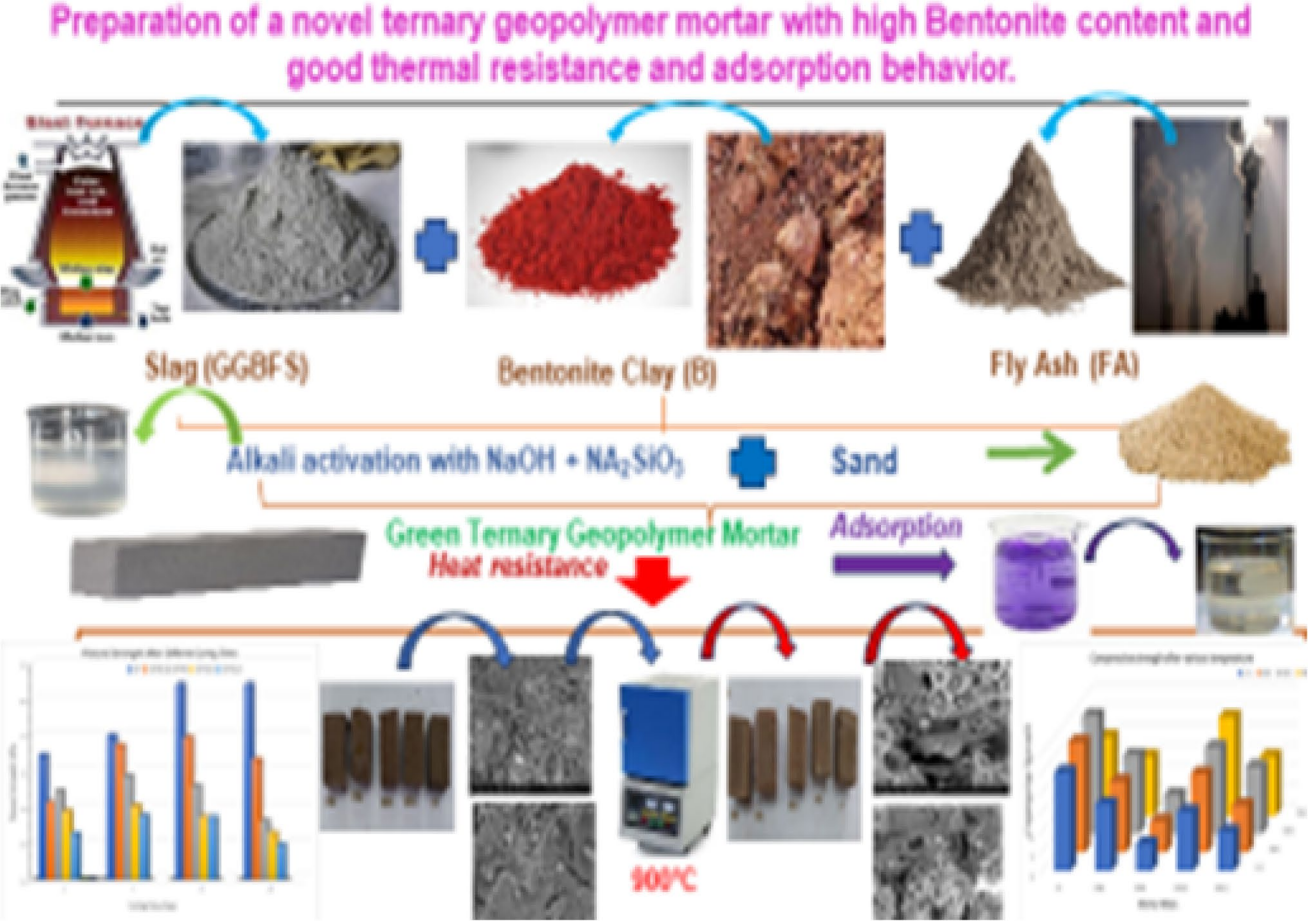
The schematic diagram of our study.

The summary of our study can be described in schematic diagram shown in Fig. 15.

## Conclusion

In this study, we examined the hydration characteristics, fire resistance, and adsorption behavior of a new green, low-cost, eco-friendly GBFS/FA-based geopolymer mortar containing different amounts of BC (6%, 8%, 10%. and 12%). The mechanical development, physical alteration, and chemical transformation of the novel ternary eco-friendly geopolymer mortar were analyzed using flexural testing, drying shrinkage, visual appearance, SEM, XRD, and FTIR. Our analysis results indicate that the newly developed ternary geopolymer mortar is an environmentally friendly alternative to conventional cement mortar by utilizing natural materials such as bentonite and industrial wastes like slag and fly ash. We found that the addition of 10% bentonite improved the heat resistance of CBFS/FA geopolymer mortar. Based on the study findings, the following conclusions are drawn.

1. The specimens of ternary geopolymer mortar that included 6% BC (SFB_6_) exhibited the best performance in terms of flexural strength.
2. The results of drying shrinkage indicate that the rate of geopolymerization and the formation of better bond strength inside the matrix at early ages for SF and SFB_6_ are higher than for other eco-friendly geopolymer mortar mixes.
3. The SEM photos reveal that SFB_6_ geopolymer mortar mix has a more uniform and denser microstructure compared to SFB_12_. This is due to the formation of aluminosilicate gel and more hydration products at an earlier stage (14 days) in the case of lower bentonite content. However, an increase in the amount of bentonite has a detrimental effect on the geopolymerization process.
4. SFB_10_ showed the best high-temperature resistance among the ternary geopolymer mortar mixes, with the lowest strength loss of (-14%) at 900 °C. In contrast, SFB_12_ showed the strongest high-temperature resistance at 600 °C, which proves that the addition of higher amounts of bentonite improves the heat resistance of the prepared eco-friendly geopolymer mortar. This indicates that the incorporation of greater amounts of bentonite can enhance the heat resistance of the prepared eco-friendly geopolymer mortar.
5. All XRD and FTIR data, both before and after heat exposure, align closely with the results of the mechanical testing.
6. Prepared geopolymer mortar mixes exhibit multilayer heterogeneous adsorption behavior for crystal violet dye removal. The adsorption results align well with the Freundlich isotherm model.

Based on our research, new geopolymer mortar mixes have been developed with heat-resistant and adsorption properties, designed for applications requiring heat-resistant cementitious materials. This environmentally friendly mortar is cost-effective, sustainable, and can be used for environmental purposes like treating industrial wastewater and facilitating eco-friendly dye removal processes.

## Data Availability

The datasets generated during and/or analysed during the current study are available from the corresponding author on reasonable request.
